# Real Time Microwave Biochemical Sensor Based on Circular SIW Approach for Aqueous Dielectric Detection

**DOI:** 10.1038/s41598-019-41702-3

**Published:** 2019-04-02

**Authors:** Amyrul Azuan Mohd Bahar, Z. Zakaria, M. K. Md. Arshad, A. A. M. Isa, Y. Dasril, Rammah A. Alahnomi

**Affiliations:** 1 Microwave Research Group, Centre for Telecommunication Research and Innovation (CeTRI), Faculty of Electronics and Computer Engineering, Universiti Teknikal Malaysia Melaka (UTeM), 76100 Durian Tunggal, Melaka Malaysia; 2 Institute of Nano Electronic Engineering, Universiti Malaysia Perlis (UniMAP), 01000 Kangar, Perlis Malaysia; 3 School of Microelectronic Engineering, Universiti Malaysia Perlis (UniMAP), 02600 Pauh, Perlis Malaysia

## Abstract

In this study, a critical evaluation of analyte dielectric properties in a microvolume was undertaken, using a microwave biochemical sensor based on a circular substrate integrated waveguide (CSIW) topology. These dielectric properties were numerically investigated based on the resonant perturbation method, as this method provides the best sensing performance as a real-time biochemical detector. To validate these findings, shifts of the resonant frequency in the presence of aqueous solvents were compared with an ideal permittivity. The sensor prototype required a 2.5 µL volume of the liquid sample each time, which still offered an overall accuracy of better than 99.06%, with an average error measurement of ±0.44%, compared with the commercial and ideal permittivity values. The unloaded *Q*_*u*_ factor of the circular substrate-integrated waveguide (CSIW) sensor achieved more than 400 to ensure a precise measurement. At 4.4 GHz, a good agreement was observed between simulated and measured results within a broad frequency range, from 1 to 6 GHz. The proposed sensor, therefore, offers high sensitivity detection, a simple structural design, a fast-sensing response, and cost-effectiveness. The proposed sensor in this study will facilitate real improvements in any material characterization applications such as pharmaceutical, bio-sensing, and food processing applications.

## Introduction

The field of material characterization in enclosures has generated a major interest in research due to its wide application in various industries. Recent developments in microwave sensor technology have heightened the need for more accurate instruments for characterizing materials in order to fulfil the demands of industry such as chemical composition analysis, food processing monitoring, agriculture-based research, pharmaceutical detection, and bio-sensing^1^. Knowledge of material properties is, of course, crucial for any understanding of proper scientific information. The dielectric characteristics of physical and chemical materials and the biomechanism of certain reactions need to be studied extensively in order to conduct an initial evaluation of materials used. Numerous studies have attempted to demonstrate, and various methods have been developed to produce high-efficiency sensors for fast detection, precise measurement, and reliable sensing capabilities^2–8^. For example, reflection, transmission/reflection, resonator, and resonant perturbation methods^9^. These types of methods can be realized with one or two-port network systems based on the specification and functionality of the sensor itself. Each method, nevertheless, is limited to specific frequency bands, selective physical properties of materials, and a narrow application by its own constraint^10,11^. Furthermore, these group of microwave methods are depending on S-parameters behaviour to detect and extract the properties of material. To be exact, the change frequency shifting of S-parameters and the E-fields distribution on sensor structure would reflect the accuracy and sensitivity of the sensor.

However, number of studies show that significant differences do exist, in a way to sense and characterize the material properties. This technique is called Surface Plasmon Resonance (SPR). Basically, SPR is an optical technique based on the detection of small changes in the refractive index on a metallic surface modified with molecular recognition materials^12^. This study demonstrated an approach in which mediator-type enzymes are used to improve SPR biosensing. Traditionally, this technique has been used widely in biosensor application, however limited on sensing larger molecules only. The structure become more complex as the optimization been made. Nowadays, there are extensive research studies on SPR technique in material characterization especially for biosensing application that explore and improving this technique^13–16^. This approach become more advanced and the innovation on magneto plasmonic structure make the sensing capability become faster and accurate. Apart from that, recent SPR development has discovered an improvement biosensing technology called SPR imaging which physically compact, reusable, inherent polarized emission and extremely high detection sensitivity. These features brings the SPR technique to the next future generation of characterization instrument^16^. Nevertheless, instead of optical technique, this manuscript focusing on microwave characterization approach as it can be integrate in sensor electronic system based on PCB integrated design such as MEMS sensor.

A large and growing body of literature has investigated microwave sensor designs, such as coaxial probes, waveguides, and dielectric resonators in order to properly characterize their material properties in terms of their polar structure and covalent bond. The drawback of such existing sensors, however, is that they are costly since most of their design structure entails a complicated measurement setup and a complex design construction^17–19^; in fact, several studies investigating the performance of the dielectric resonator sensor confirmed this drawback^20,21^. Typical design complexity is involved in order to achieve a high level of efficiency for better performance, without considering the length and complexity of product realization procedure, nor cost consumption. Similar disadvantages are associated with the production of the coaxial probe method^22^, where the non-planar geometrical design necessitates a high manufacturing cost and where the volume of liquid must large enough for the sensor to be completely submerged in order to gain any interaction between the electromagnetic wave signal and the dielectric properties of the sample.

Therefore, a planar structure with a specific topological approach is proposed in this paper, in order to overcome the drawbacks of existing sensors. The proposed sensor is purposely designed to be more compact, simpler, to have a lower manufacturing cost, and to be easier to fabricate. Nevertheless, a common concern is that most planar sensors suffer from poor quality factor (Q-factor) and high measurement errors, which limit their usage in many applications, as is evident in many existing designs^23,24^. Hence, new alternative techniques are required to overcome the drawbacks of current planar sensors. Although there are numerous research studies on waveguide microwave planar sensors^23–25^, studies on circular substrate-integrated waveguide (CSIW) technology used in biochemical sensors are rarely to found in terms of aqueous characterization.

With advances in substrate-integrated waveguide (SIW) technology in the last decade, it is now possible to develop planar sensors with the level of functionality previously only earmarked for conventional waveguide structures. Unfortunately, the complexity of design significantly limits their use and leads to the use of planar resonant sensors, which are generally compact, cost-effective, and easier to manufacture. SIW resonators are still, nevertheless, a relatively new class of planar resonant devices, which offer higher quality factors in comparison to other planar devices. The improvement has been reported in^25–27^, where the author utilizes a rectangular SIW to investigate the properties of an aqueous solution with a one port network analysis. The author^27^ presented a high sensitivity sensor boasting micro-volume measurement, but which provided limited specificity and applicability for such a sensor. The authors in^25^, nevertheless, demonstrated accurate measurements with the simple and low-cost design, but a large volume of the sample was required for an immersed method of measuring.

In general, as observed from prior studies, it is difficult to obtain optimum performance using the planar design structure. Another motivation for this study is, therefore, to propose a microwave planar biochemical sensor based on CSIW, in order to enhance and demonstrate the efficacy of the concept of precise measurement using planar structure. This sensor provides a high-quality factor which is better than 400 in order to minimize measurement error detection; in fact, the overall quality factor is said to be better, based on comparative studies in the literature^19^. The proposed sensor is not only better in terms of performance, but the physical structure is more reliable, simple, and less expensive. It also requires only microliter measurement and is compatible with integration in other electronic components. The basic idea and theory are the same as presented by the author in^28^. Although this paper applies reflection measurement of a rectangular resonator instead of transmission measurement of a circular resonator, the measurement technique is quite similar. The field distribution pattern and flux density for circular shapes are better than for rectangular structures with the same cross-sectional area^29^. As a thorough search of the relevant literature yielded no other similar examples, this is the first time that the two-port network of CSIW structure is being proposed as a microfluidic sensor. It is true that a few CSIW resonators have been proposed to date, but none of them have been demonstrated as a microfluidic biochemical device.

## Working Principle and Sensor Design

### The circular substrate integrated waveguide resonator

The SIW structure implemented in this work was a circular waveguide formed by two rectangular conductor planes, which were separated by some dielectric material. The dielectric and conductor were emulated by plated through-hole vias. The circular SIW geometrical structure was designed by choosing appropriately spaced via holes with the same diameter as a guided wave pattern; designed in this way for minimum radiation loss^9^. The electric field distribution in the waveguide structure depended on the spacing between the vias excitation. This spacing actually controlled the standing wave pattern through the medium. If the vias were spaced too far apart, the isolation properties of the SIW would be compromised. The limitation of mode propagation could be set according to the leakage potential of the resonator sensor. The patterns of TM and TE modes needed to be strictly understood in order to choose a correct transverse mode for the design structure^9^.

**Figure 1:**
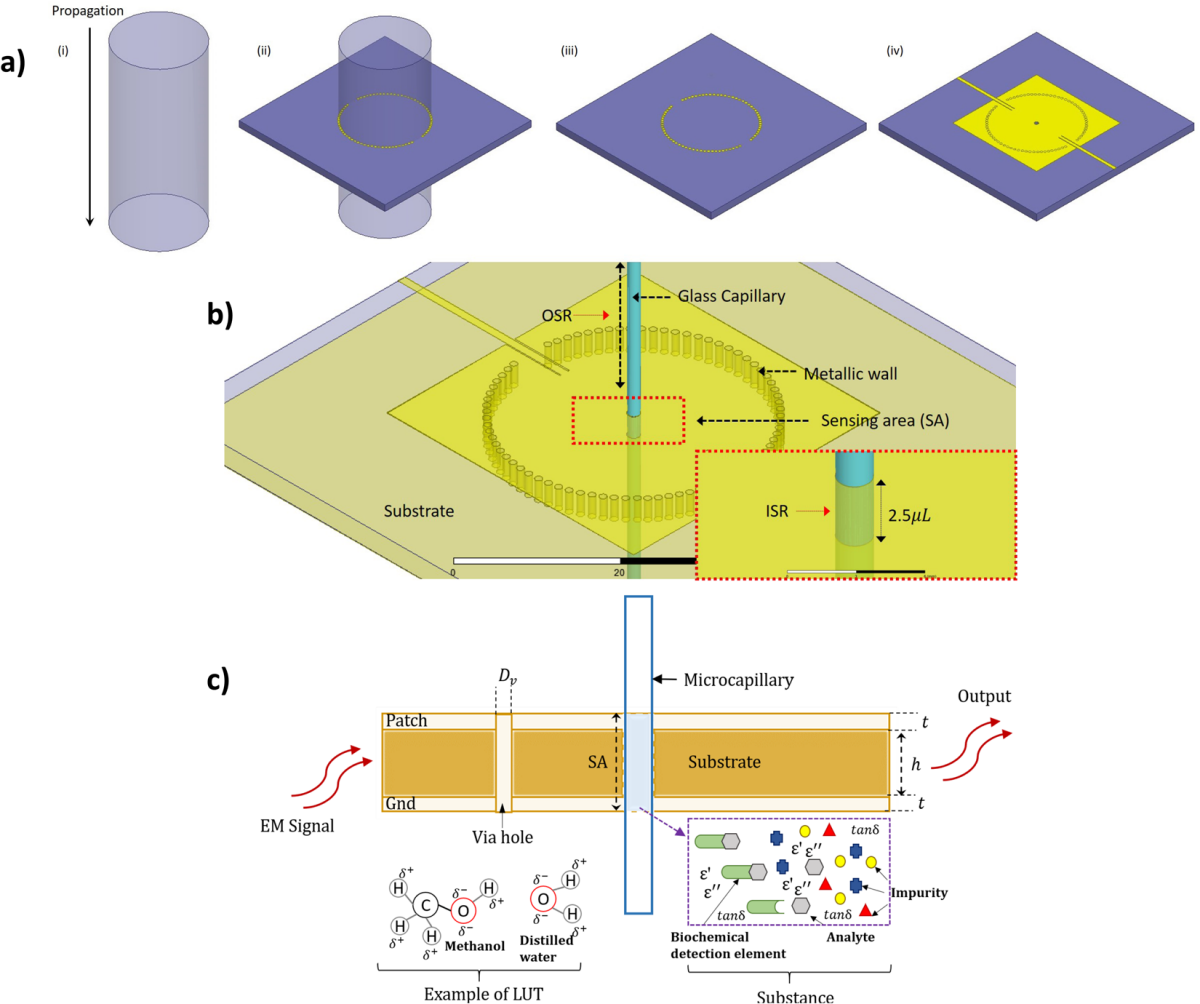
Schematic illustration of (**a**) Creation of CSIW resonator from the single substrate on TM mode. (**i**) Cylindrical schematic waveguide structure propagating TM modes, (**ii**) *TM*_*mn*0_ substrate thickness cavity manufactured from the waveguide, (**iii**) SIW with arrays structure of via-holes, (**iv**) CSIW complete structure with waveguide on *TM*_*mn*0_ modes. (**b**) Close up view of biochemical sensor integrated with glass microcapillary channel as sensing area (SA) with the trace volume of sample (**c**) Working principle detection.

The schematic hollow metallic waveguide crossed the substrate of the CSIW structure perpendicularly, using TM modes notation, as shown in Fig. 1a(i). In Fig. 1a(ii), in contrast, the TM modes occurred when the right angle between the substrate and the schematic waveguide were aligned, in which the TM substrate thickness cavity was manufactured from the waveguide. Subsequently, the current line of TM mode waveguides was formed along the waveguide structure and went through the arrays of via holes, as indicates in Fig. 1a(iii). In this way, TM mode could be set up. The height of the cavity was synchronized with substrate thickness via the schematic waveguide to produce its identical structure^27^. Figure 1a illustrates the overall design of the CSIW. A detailed explanation of the development of a CSIW resonator from a single substrate in TM mode is shown in Supplementary Information 1.

### The sensing principle

The hole structure at the center of the device cavity interrupted the transverse surface currents in the metallic wall, thereby forming an electromagnetic (EM) pattern, which depended on the TM mode magnitude. A microfluidic subsystem, consisting of a glass capillary and a channel slot with a diameter of 1 mm, was subsequently integrated into the center of the sensor layer, as shown in Fig. 1(b). The rubber band stopper, acting as an aqueous channel holder, was then placed on top of the microfluidic subsystem to condense the aqueous sample inside the glass capillary. The glass capillary was transversely located at the center of the proposed sensor where the propagated EM near-field was concentrated, in order to achieve better sensing precision and sensitivity^26^. The operation sensing principle was to track any changes in resonant frequency and losses in the sensing slot. As a result, the interaction between the propagated EM near-field and the condensed aqueous sample in the glass capillary could be resolved.

The maximum number of electrical fields were only concentrated at the channel slot cavity of the device, which measured 2.5 microliters (*μL*) volume of liquid at a time. A close-up view is shown in Fig. 1(c) to indicate the sensing area. This area comprised the sensitive biochemical element, the sensing area (SA) or indicator element, and related electronic components or signal processors which mainly accountable for the demonstration of the findings in a user-friendly approach. The geometrical parameters of the waveguide structure and resonator device could then be determined by the desired resonant frequency of the dominant mode. The design specification is discussed in the following section and a detailed explanation of the physical dimensions of the proposed structure is illustrated in Supplementary Information 2.

## Design of the Biochemical CSIW Resonator Sensor

**Figure 2:**
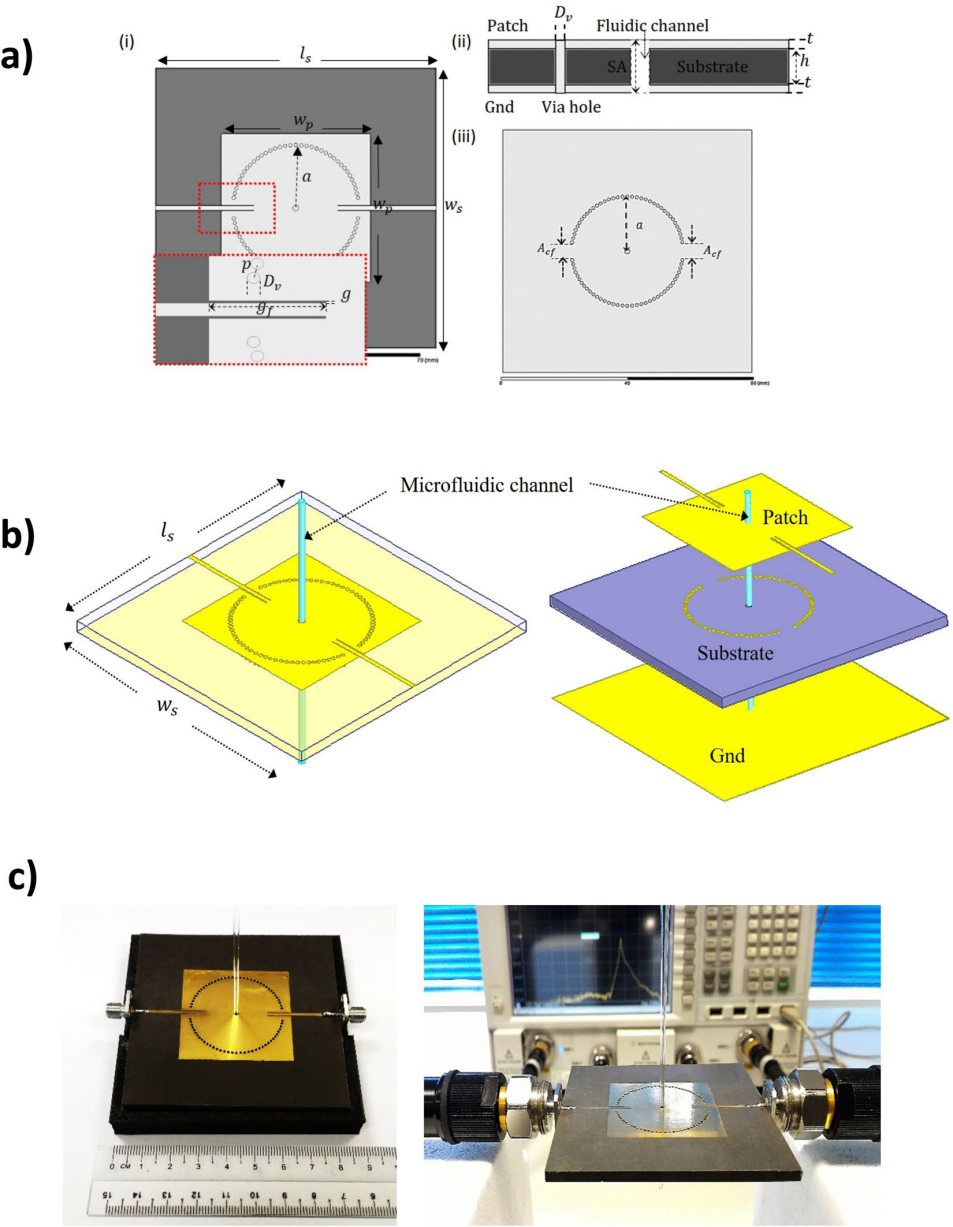
Design of planar resonator biochemical sensor: (**a**) The overall design for planar resonator biochemical sensor without the microfluidic channel. (**i**) Top view with close up view to feed line and via hole structure. (**ii**) Side view showing the substrate, ground, and dimension of the sensing area, (**iii**) Bottom view showing the grounding structure from plated through hole (PTH) technique. (**b**) Layout and layer structure of the proposed CSIW resonator sensor with perspective view of transparent substrate. (**c**) The final structure of fabricated biochemical sensor for dielectric properties detection with measurement system.

The proposed CSIW resonator sensor was to operate at 4.4 GHz with the first mode of the resonant frequency and the sensor measured, at a broadband frequency, within a range from 1 GHz to 6 GHz. Roger Duroid RT5880 was employed, with a dielectric constant of *ε*′ = 2.2 and a tangent loss of *tanδ* = 0.0009. The thickness of the substrate was 3.175 mm, on account of the large scale of the sensing region. The dimensions of the resonator were: *t* = 0.035 *mm*, *l*_*s*_ = 79.92 *mm*, *w*_*s*_ = 79.92 *mm*, *a* = 17.59 *mm*, *w*_*p*_ = 42.42 *mm*; and the diameter of via hole was *D*_*v*_ = 0.5 *mm*, with the distance between the via hole being *p* = 1.6 *mm*, as shown in Fig. 2a. In fact, all geometrical dimensions of the proposed sensor are clearly illustrated in Fig. 2a(i),(ii),(iii), respectively. The sensor can be easily scaled and, therefore, re-designed in order to work in other frequency regions. The resonator is frequency selective, but many sensors are not required to arrive at spectroscopic results since the measurements take place within a broad frequency range as the dielectric properties of the material depend on frequency and temperature. Regarding the device material, the substrate has a low radiation loss and is easy to fabricate with less technical procedures. Most importantly, since this research deals with liquid materials, the material exhibits low moisture absorption and is, therefore, suitable for solvent measurement, even though there is a microfluidic channel for sample measurement.

Both coupling gaps at the feedlines (*g* = 0.2 *mm*) were implemented to control capacitance strength. There were 74 plated through hole (PTH) vias. These were circular-shaped, with grounding from the top patch to the ground as a waveguide excitation. The coupling and feedline area, *A*_*cf*_ controlled the EM flow to constrain the wave propagating into the device. This topological structure offered an extremely high density of electric flux to gain optimum sensitivity measurement. This type of CSIW resonator sensor had an overall dimension of 79.92 *mm* × 79.92 *mm* × 3.245 *mm* (*l*_*s*_ × *w*_*s*_ × *h*), after optimization, described in Fig. 2(b). The parameters study was then performed to optimize the overall dimension in order to minimize the bandwidth at operating frequency.

The CSIW planar resonator sensor was fabricated and tested with a microwave frequency band after optimizing the performance response and ensuring the device specifications were met. The proposed design procedures involved five standard processes, which involved taking advantage of printed circuit boards (PCBs). The fabrication process entailed the generating mask transparency, photo exposure processes, etching in a developer solution, etching in ferric chloride, soldering the port, plating the through-hole (PTH) vias, and drilling the center-holes for a microfluidic channel. The resonant frequency was shifted with the presence of aqueous solution into the microfluidic channel, which corresponded to the maximum concentrated electric flux density. The proposed CSIW structure had only 3 layers. A full-wave simulation was performed by the high-frequency structural simulator (HFSS) and measurements were taken using the Vector Network Analyser (VNA) to measure and validate the S-parameter response. Figure 2(c) illustrates the fabricated prototype of the proposed CSIW resonator. Top patch and ground layers were fabricated through a PCB etching process and the middle layer was made-up by drilling a 1 *mm* diameter hole for the microfluidic channel slot and the PTH grounding vias. The sensor was then laminated with adhesive bonding gold film to both protect the surface layer from oxidization and for better conductivity. The results are discussed in the following section.

## Measurement and Results

**Figure 3:**
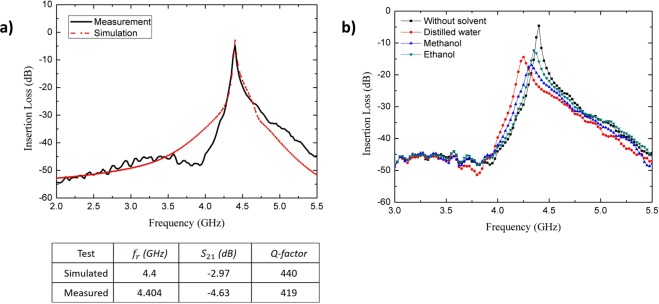
(**a**) Simulated and measured results of the resonant frequency response of CSIW sensor. (**b**) Measurement of the common solvents using CSIW resonator sensor at 2.5 µL volume of sample.

In order to characterize the aqueous sample using the CSIW topological approach, a mathematical model was required to determine the dielectric properties of the test sample. The aqueous solution was modeled into the numerical solver by inserting the standard properties of each solvent. The electromagnetic solver Ansys HFSS^30^ was executed for numerical simulation. A real-time domain simulation was used to optimize the physical and electrical parameters of the proposed sensor. The wave propagation of the EM signal was excited by means of two waveguide ports (each having a 50 Ω impedance). An identical agreement was observed between simulated and measured results at 4.4 GHz, as presented in Fig. 3(a). The simulated resonant frequency, insertion loss, and the quality factor of the proposed sensor in the unloaded condition were found to be 4.4 GHz, −2.97 dB and 440, respectively. The experimental measurement results of those three parameters were found to be 4.404 GHz, −4.63 dB and 419, respectively. Accuracy, which depended on quality factors and the measurement of error detection, could be validated based on the standard value of complex permittivity at a specific frequency and temperature. A critical evaluation was made to ensure that the measurement results were parallel to the theoretical concept. The loaded Q-factor and the resonant frequency were reflected in the bio-sensing characteristic since the loaded Q-factor referred specifically to the performance of the sensor, and the shifting of the resonant frequency referred to the perturbation of electric flux with the presence of samples. This factor might also render significant evidence concerning whether the aqueous bio-chemicals bind with or unbind from each other. For different aqueous samples possess specific biochemical properties, which reflect strong or weak bindings, whereas the shifting of resonant frequency reflects inter-biochemical coupling.

Several common solvents were tested (distilled water, ethanol and methanol) to measure reliability and validate sensor efficiency. The concentration of the aqueous sample was 100% pure solution with 2.5 µL volume at a time. An empty glass capillary with a relative permittivity of 5.5 was integrated with the CSIW sensor as a microfluidic channel. The glass capillary was placed at the center of the waveguide structure, which was concentrated at optimum density for an electric flux distribution. The common solvents were measured (magnitude and phase) from 1 to 6 GHz using the Agilent Vector Network Analyzer. It was necessary for the room temperature to remain constant so that any unwanted condition errors during the measurement could be avoided. The resonant frequency of each sample (distilled water (H_2_O), ethanol (C_2_H_6_O) and methanol (CH_3_OH)) had a different dielectric constant (80.1, 24.5 and 32.7 respectively), which contributed to the shifting of frequency due to the use of the perturbation method, as presented in Fig. 3(b). The polar nature of the aqueous solution carried valuable information from the transmission and reflection coefficient of the electromagnetic waves. Hence, the behavior of resonant frequency shifting and the quality factor played an important role in extracting the sample properties.

The values of the resonant frequency, insertion loss (*S*_21_) and bandwidth of frequency shifting (Δ*f*) were generated from the transmission coefficient data and were then analysed for the derivation of a mathematical model with the Curve Fitting (CF) technique as shown in Table 1. The software *Origin pro 8* was employed to generate the imperative equations using the CF technique in order to determine the complex permittivity of the LUT. The resulting fitting equation was generated based on the least error between the selected profile and the collected datasets of numerically obtained data.

**Table 1:** The transmission coefficient changes with the presence of sample.

Aqueous Sample	Resonant Frequency (GHz)	*S*_21_(dB)	Δ*f*(GHz)	Ideal real permittivity (*ε*′)^27^
Without capillary	4.4000	−2.9717	0	—
Air	4.4035	−4.6336	0.0035	1.00059
Ethanol	4.3470	−12.3888	0.0530	24.5
Methanol	4.3300	−16.9336	0.0700	32.7
Water	4.2510	−14.4369	0.1500	80.1

### Determination of the real part permittivity

**Figure 4:**
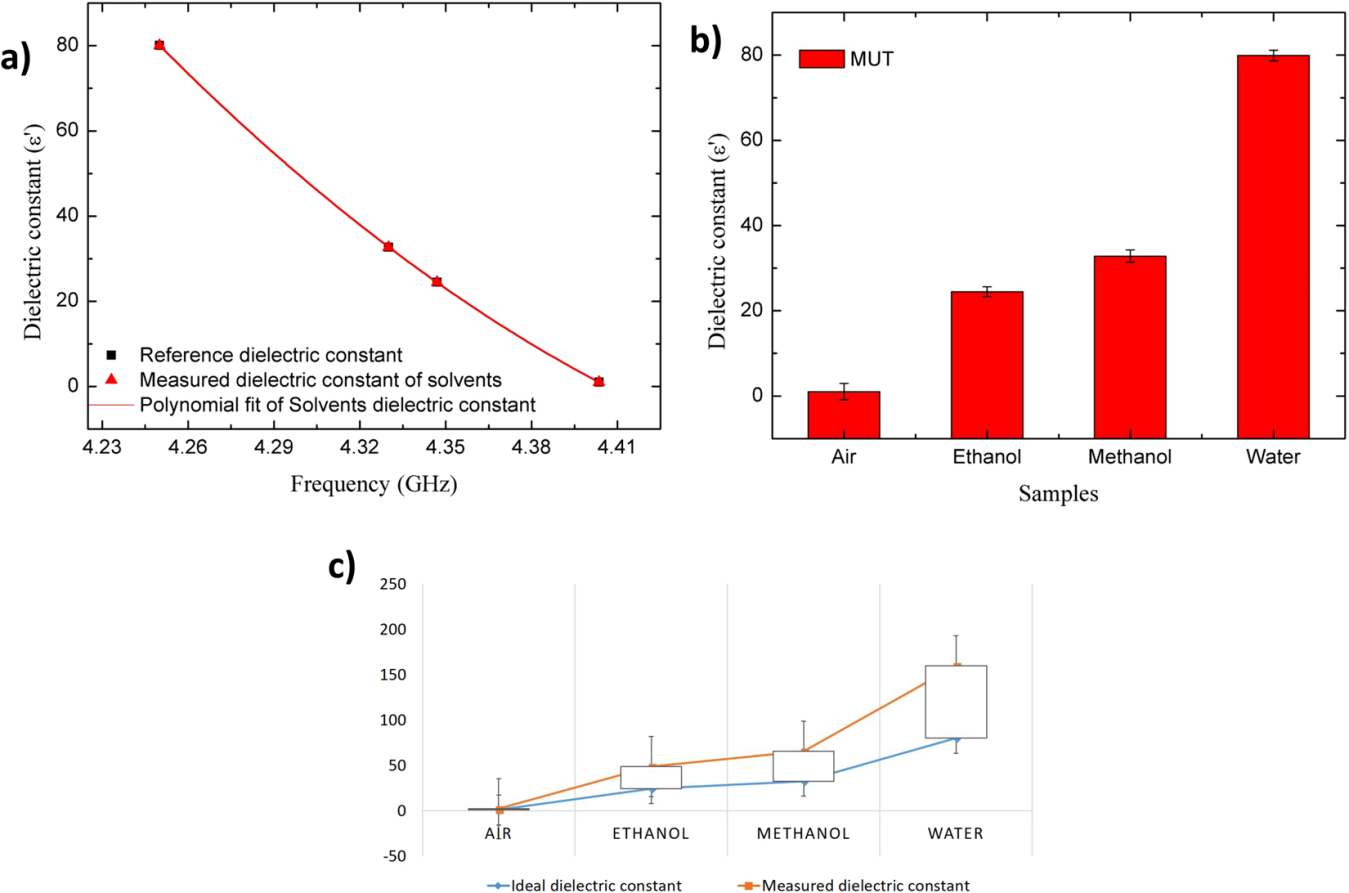
Dataset analysis (stacked line) of dielectric constant using proposed sensor (**a**) Polynomial curve fitting of ideal and measured dielectric constant to develop formulation (**b**) Percentage errors with respect to measured solvents (**c**) Standard error trend line for both datasets.

The following approach was employed to determine the real part of the complex permittivity (*ε*) in terms of the resonant frequency shifting. The response of the sensor covered with different aqueous solvents was simulated using HFSS. The liquids were simulated taking into account their dielectric characteristics obtained using the dielectric probe kit. The shifting of the resonant frequency, (Δ*f*) extracted from the measurement data sets, was then plotted with the corresponding ideal permittivity (*ε*′) of the Liquid under Test (LUT), as presented in Fig. 4. It can be observed from Fig. 4(a) that the variation of *f* and *ε*′ is inversely proportional and the dielectric constant of the LUT is numerically expressed using polynomial fitting technique with 2^nd^ degree function modelling as stated in equation (1). The numerical modelling has been generated from the experimental data and fitted with the reference dataset. 2^nd^ degree function is significant to dealing with the experimental data as it originated the highest R2.

1 $$\varepsilon ^{\prime} =10667{f}^{2}-97491f+22247$$

The above formulation was established using CF technique, to determine the real part permittivity of the sample, where *ε*′ is a dielectric constant (real permittivity) and *f* is a resonant frequency of the presence solvents. At this point, the dielectric constant of any liquid (unknown permittivity) sample could be derived from that equation. The results were critically compared with the ideal permittivity of liquid solvents and commercial sensor measurement (Agilent 85070E Dielectric Probe Kit)^31^ to validate the data. The percentage error function and the standard error trend line of dielectric constant are shown in Fig. 4(b,c), respectively. Scattering parameters of a microwave sensor can be influenced by electric and magnetic properties of its near environment. This interaction can be reflected in forward transmission gain (*S*_21_) of a resonator. Presence of analyte or any impurities within the medium inside the microfluidic channel affects the amplitude and the resonant frequency of the dedicated device. The equation (1) is only applicable for the real part permittivity analysis since the fitting technique is dependent on the least error dataset from both standard ideal and measurement values that coming from the tested device. However, the generated equation can be applying to all types of liquid sample to characterize its properties.

### Determination of the loss tangent (tanδ) and imaginary part permittivity

**Figure 5:**
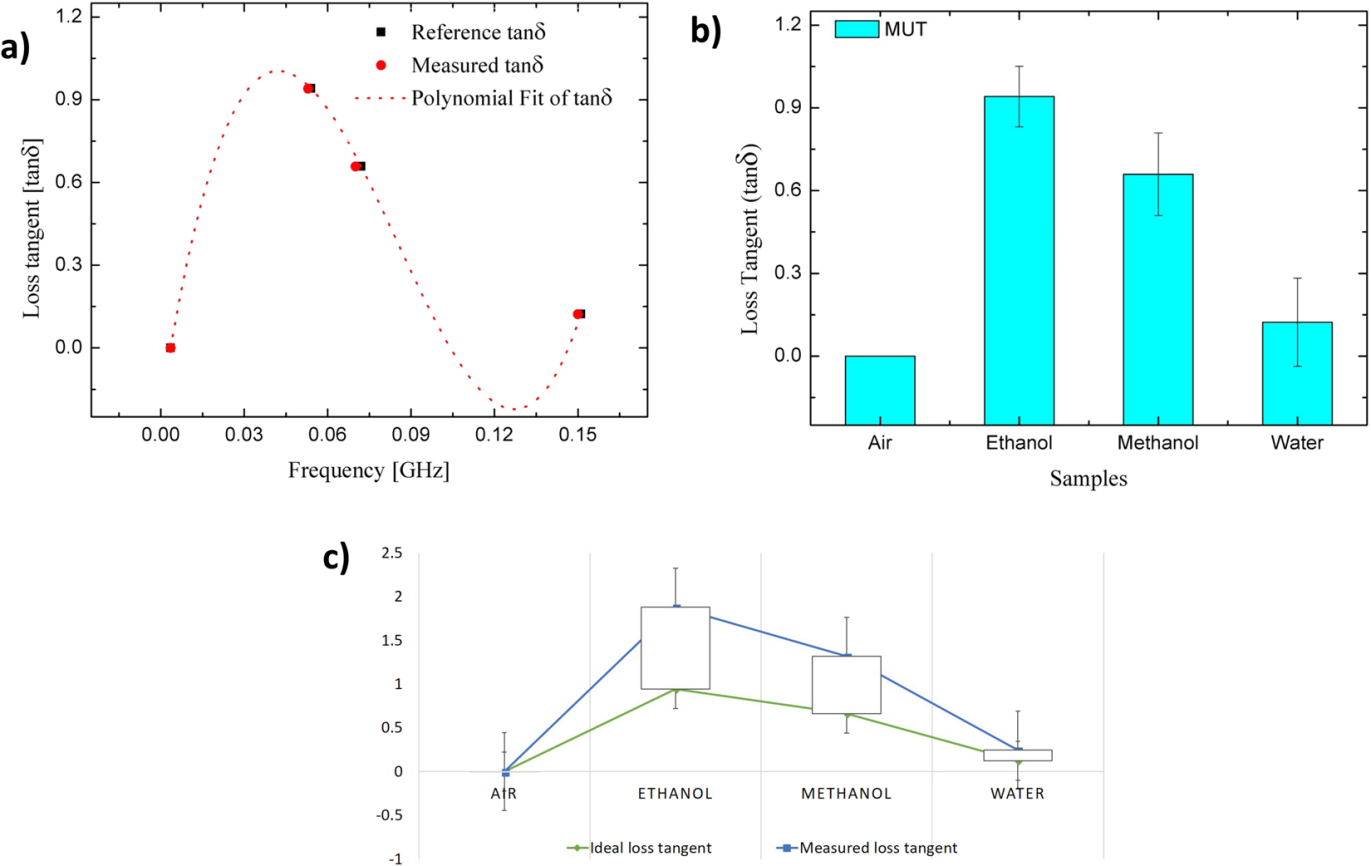
Dataset analysis (stacked line) of loss tangent using proposed sensor (**a**) Polynomial curve fitting of ideal and measured loss tangent to develop formulation (**b**) Percentage errors with respect to measured solvents (**c**) Standard error trend line for both dataset.

A similar analysis was executed to establish a mathematical model for scheming the loss tangent (tanδ) and imaginary part (*ε*″) of the complex permittivity. The magnitude of transmission coefficient (Δ*f*) between the unloaded and loaded sensor was observed and recorded. A graph was plotted from these particular data sets with respect to an ideal loss tangent as shown in Fig. 5(a). From this figure, it may be noted that the variation of the *tanδ* with Δ*f* was not linear. However, the relationship between both parameters could be defined in 3^rd^ order polynomial expression as the best-fitting graph to generate an accurate numerical model, as stated in (2), instead of in 2^nd^ order function. The accuracy was due to the graph pattern between both results (ideal value and measured value). The expression was developed by a curve fitting (CF) technique and validated through a comparison between the ideal values and a commercial sensor measurement. The percentage error function and the standard error trend line of loss tangent are shown in Fig. 5(b,c), respectively.

2 $$tan\delta =4349.9{(|{\rm{\Delta }}f|)}^{3}-1085.6{(|{\rm{\Delta }}f|)}^{2}+67.266(|{\rm{\Delta }}f|)-0.2223$$

where $tan\delta$ is a loss tangent and Δ*f* is a frequency shifting from perturbation response of a sample of molecular structure. After establishing the numerical expressions (1) and (2) for calculation of the real part permittivity and loss tangent, the imaginary part of the sample could then easily be found by derivation of all those three relationships, referred to in Supplementary Information 3^32^.

In order to characterize the properties of sample (even unknown material) the derivation of mathematical model is one of the best way. One way to do this is to use fitting technique where generate a formula that best fits a given set of data. Either linear or non-linear, these functions give the best least‐squares fit to base data. The values of parameters that yield the best fit function can generate equation which is highly accurate for specific parameter values as in this case, the real part permittivity and the loss tangent. Equations (1) and (2) with Figs 4(a) and 5(a) shows how the fitting data been done. Both ideal and measured datasets are fitted using 2nd and 3rd order polynomial functions, respectively.

### Data Analysis of complex permittivity

After the fabrication process, all the measurements were performed using the Agilent Vector Network Analyser. Several common solvents were characterized using the proposed sensor and commercial sensor (Agilent 85070E Dielectric Probe Kit). The transmission coefficient data for all cases were verified using VNA with different dielectric samples. The recorded resonant frequencies obtained using the CSIW sensor for air, ethanol, methanol and distilled water were 4.4035, 4.347, 4.33 and 4.251 GHz, respectively, while the insertion losses were −4.6336, −12.3888, −16.9336 and −14.4369 dB, respectively. The measured resonant frequency and the bandwidth magnitude of the transmission coefficient for each aqueous sample were then form-fitted into the established mathematical model and the corresponding dielectric constant, *ε*′ and loss tangent, *tanδ* were calculated using Equations (1) and (2), respectively, as tabulated in Table 2.

**Table 2:** The comparison of the LUT dielectric properties with different measurement methods.

Methods	Dielectric properties	LUT			
		Air	Ethanol	Methanol	Water
Ideal Values^27^	*ε*′	1.00059	24.5	32.7	80.1
	*ε*′	0	23.0545	21.5493	9.8523
	*tanδ*	0	0.941	0.659	0.123
Agilent 85070E Sensor^31^	*ε*′	1.0012	24.4057	32.5538	75.8915
	*ε*′	0.0065	22.2657	19.4529	19.6661
	*tanδ*	0.00649	0.9120	0.5976	0.2590
	diff (%)	0.06	0.38	0.45	5.25
Proposed Sensor (CSIW)	*ε*′	1.01	24.46	32.84	79.91
	*ε*′	1.92 × 10^−5^	22.9924	21.6087	9.7490
	*tanδ*	1.9 × 10^−5^	0.940	0.658	0.1228
	diff (%)	0.94	0.16	0.43	0.24

The measured results were also compared with the ideal existing datasets in the literature, at room temperature (25 °C). A constant temperature must, of course, be ensured to avoid misleading information during the measurement process. The S-parameter data-set from the Agilent 85070E dielectric sensor were also presented and compared in Table 2 for validation purposes. For the precise measurement of the proposed sensor, the percentage error was calculated and shown in column 12 of Table 2, with respect to ideal values. It can be concluded that the results of the complex permittivity and loss tangent measurements using the CSIW topology sensor were in fact in positive agreement. It may be noted that the complex permittivity measurement of both the commercial sensor and the proposed sensor was possible with an average error of ±1.54% and ±0.44%, respectively.

Errors obtained from the measurement results could be attributed to a number of factors, such as a geometrical mismatch between the simulated and prototype models, or possible biochemical reactions between the aqueous sample and the surrounding atmosphere. The geometrical errors could be mitigated by using high precision instruments, which are in fact widely used in the sensor fabrication industry, while a fraction of errors, which were present due to the chemical reactions of the sample, might be numerically modeled, a task beyond the scope of this study. A comparison between previous works and the proposed sensor in this study is presented in Table 3. The percentage errors are compared between several existing studies based on planar resonator sensors with several common solvents. In order to prove the reliability of the proposed sensor, the influence of aqueous volume and concentration could also be taken into account, along with a critical analysis, as explained in the next sub-section.

**Table 3:** A comparison between previous research works and the proposed sensor.

Microwave Planar Resonator Sensor		Solvents		
		Ethanol	Methanol	Water
MSIW^11^	Ideal *ε*_*r*_	24.50	32.70	80.10
	Measured *ε*_*r*_	23.33	—	74.86
	Error (%)	5.0	—	7.0
SIW Cavity^25^	Ideal *ε*_*r*_	24.50	32.70	80.10
	Measured *ε*_*r*_	25.96	—	—
	Error (%)	5.62	—	—
DSRR^33^	Ideal *ε*_*r*_	24.50	32.70	80.10
	Measured *ε*_*r*_	24.32	32.50	78.36
	Error (%)	0.74	0.62	2.22
CSIW[Proposed]	Ideal *ε*_*r*_	24.50	32.70	80.10
	Measured *ε*_*r*_	24.46	32.84	79.91
	Error (%)	0.16	0.43	0.24

### Aqueous volume analysis

**Figure 6:**
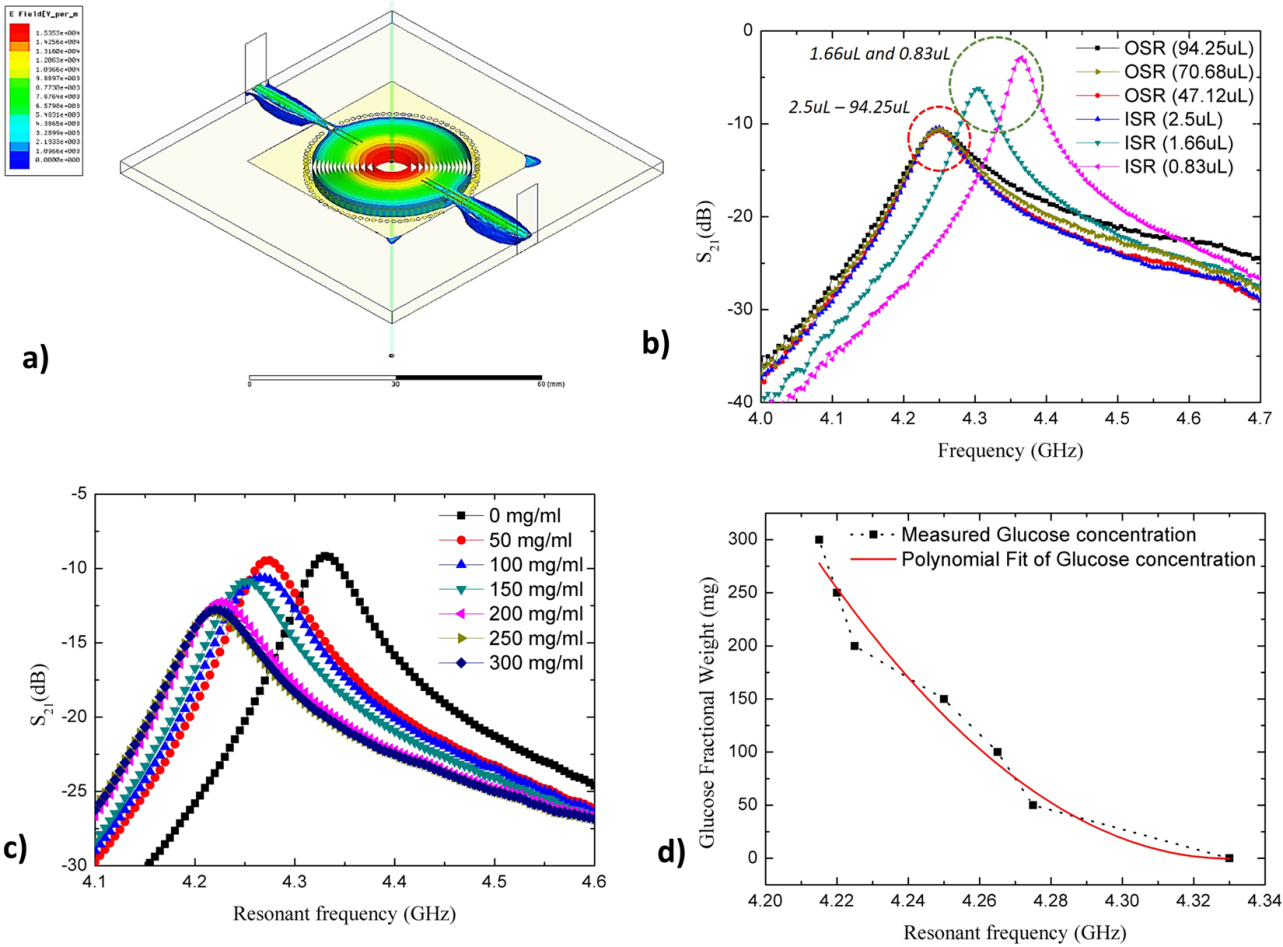
Reliability analysis of proposed sensor (**a**) The fields distribution of the proposed structure. (**b**) The variation of aqueous sample using CSIW sensor (**c**) Liquid mixture analysis of S_21_ plotting for distilled water and glucose (C_6_H_12_O_6_) mixture concentrations (**d**) Fitted plotting of glucose fractional weight against resonance frequency.

Variation of aqueous volume analysis is an important factor in proving the reliability of the planar structural sensor and the accuracy of the characterization measurement using the proposed sensor. For liquid volume will, of course, affect the resonant frequency in the sensing region, as shown in Fig. 6(a). This is due to the perturbation of a high density of electromagnetic fields distribution with the polar nature of chemical and dielectric properties of LUT. Apart from that, the presence of *TM*_01_ mode of the E-fields notation on proposed sensor technically optimized the maximum fields on the centre sensor structure and at the same time improved the sensitivity and measurement accuracy. At this rate, the LUT properties can be characterize with minimum trace amount of volume (2.5 uL). *TM*_01_ mode is implemented on this design due to high centre flux density. The understanding of transverse mode properties is significant due to the sensor behaviour as elaborate in Supplementary Information 1. Also, the sensing area depends on substrate thickness where electric fields propagate through the structure to characterize the LUT properties. In Fig. 6(b), it may be observed that the slope of the plotted graph is influenced by the volume of LUT. It is also noticeable that the slope of the curve remains constant for a sample volume greater than 2.5 µL. This actual behavior may be observed from the four plotted lines corresponding to the sample volume of 2.5 μL until 94.25 μL, where all curves overlap each other. However, if the volume is less than 2.5 μL, the resonant frequency will be shifted, as shown in Fig. 6(b) and Table 4. These scenarios happened due to sense of region with respect to electric fields distribution. The E-fields exists on sensing area (SA) as shown in Fig. 1(c). Whenever the LUT volume change in sensing region (SA), the perturbation of frequency response would change accordingly. However, if the LUT volume exceeding the maximum SA region, the frequency would only respond to the maximum volume (2.5 *µL*). This is indicated the prototype reliability from volume perspective. For example, the frequency response shifted to 4.305 GHz and 4.365 GHz when the volume was varied to 1.66 μL and 0.83 μL, respectively. Therefore, the minimum volume for accurate measurement was validated at 2.5 μL.

**Table 4:** The liquid variation analysis.

Liquid variations	Volume (*μL*)	Frequency (GHz)	*S*_21_ (dB)
Out of Sensing Region (OSR)	94.25	4.25	−10.72579
	70.68	4.25	−10.53995
	47.12	4.25	−10.63813
In the Sensing Region (ISR)	2.50	4.25	−10.44177
	1.66	4.305	−6.22288
	0.83	4.365	−2.90798

### Aqueous Concentration Analysis

The CSIW sensor demonstrated aqueous quantification by measuring the sample mixtures of distilled water and glucose (C_6_H_12_O_6_), with both compositions varying from 0–300 mg by volume (100 ml). An increment of glucose weight by 50 mg, corresponding to an equal volume of distilled water for each measurement step, was added. A similar analysis can be seen in several existing studies on biosensors, which demonstrated the detection of glucose for therapeutic agent^22^. Zero milligram glucose constituent in the sample mixture indicated 100% distilled water. Figure 6(c) and Table 5 shows the measurement result representing the reflection coefficient (*S*_21_) of the different aqueous concentrations.

**Table 5:** The aqueous concentration analysis.

Fractional weight of glucose (C_6_H_12_O_6_) (mg)	Resonance frequency shifted (GHz)	Insertion loss shifted (dB)
0	4.33	−9.15547
50	4.275	−9.46245
100	4.265	−10.65061
150	4.25	−10.87486
200	4.225	−12.26541
250	4.22	−12.86076
300	4.215	−12.94661

Varying sample mixture concentrations resulted in a comparable change in resonant frequency and amplitude signal. This change was generally due to a high loss liquid having more perturbation influence on the resonated near-field than a lower loss liquid, thereby resulting in a lower resonant frequency. Therefore, by preparing 100% distilled water and causing an increment of each measurement step upwards by 50 mg weight, the resonant frequency correspondingly shifted downwards as more glucose by weight dominated the test sample. This is illustrated in Table 5 and Fig. 6(d), with datasets of the concentration model, being developed by a 2^nd^ function polynomial fitting curve technique. As a result, this resonator circuit model was found to embody the behavior of this particular mixture and could subsequently be used to investigate the composition of any given distilled water–glucose (C_6_H_12_O_6_) mixture. The estimation equation characterizing the concentration measurement was given as

3 $${W}_{f}=20963{f}^{2}-181549f+393072$$

where *W*_*f*_ is the fractional weight of glucose and *f* is the measured resonant frequency.

## Conclusion

A new class of microwave biochemical sensor based on circular substrate integrated waveguide (CSIW) topology has been successfully designed and validated. The proposed sensor works on 4.4 GHz. Therefore, a high-accuracy measurement and high-sensitivity sensor, with respect to the complex permittivity and loss tangent of microfluidic solvents, is presented. A mathematical model has been developed for determining the dielectric constant and loss tangent of the LUT. The average error detection had a lower percentage value of ±0.44% as compared with the ideal permittivity of aqueous samples. The maximum relative error detection is 0.94%, which boasts better accuracy compared to existing resonator sensors with more than 400 of the Q-factor. The polynomial curve fitting method was applied for the determination of the dielectric properties of the material. The proposed CSIW sensor was found: to be more accurate; to be easier to fabricate; to have a lower cost of manufacturing; to have a higher detection response; and to be compatible for integration with other electronic components in microwave and RF engineering. These results can contribute more widely to intrinsic biochemical information, with the potential to be extended for various applications. In particular, the biochemical parameters of the resonant frequency set in this study can offer the outstanding advantage of users being able to transfer this valuable information by means of a remote sensing technique.

## Supplementary information

Supplementary Information
