# Supplementary material for: Real Time Microwave Biochemical Sensor Based on Circular SIW Approach for Aqueous Dielectric Detection
Source: Sci Rep. 2019 Apr 2;9:5467. doi: 10.1038/s41598-019-41702-3 (PMC6445140; doi:10.1038/s41598-019-41702-3)
Supplement: Supplementary file 1 — Supplementary Information [file 41598_2019_41702_MOESM1_ESM.pdf]

Real Time Microwave Biochemical Sensor Based on Circular SIW Approach for Aqueous Dielectric Detection

Amyrul Azuan Mohd Bahar[a], Z. Zakaria[a], M.K. Md Arshad[b, c], A.A.M. Isa[a], Y. Dasril[a], Rammah A. Alahnomi[a]

Supplementary Information 1

In this work, TM modes were applied due to the existence of the TM mode in circular shape excitation to the specific electric flux pattern. The wave equation in circular shape coordinates is given by

$$\left(\frac{\vartheta^2}{\vartheta\rho^2}+\frac{1}{\rho}\frac{\vartheta}{\vartheta\rho}+\frac{1}{\rho^2}\frac{\vartheta^2}{\vartheta\theta^2}+k_c^2\right)e_z=0 \tag{1}$$

E_z is the field component of transverse mode for TM mode where $E_z(\rho,\theta,z)=e_z(\rho,\theta)e^{-j\beta z}$, and $k_c^2=k^2-\beta^2$. Since this equation is identical to condition stated in (Pozar, 2005). The general solutions are the same. Thus, from the equation (2), the e_z can be found as (3) and the first few values are listed in Table 1 (Chen et al., 2004).

$$h_z(\rho,\theta)=(A\sin n\theta+B\cos n\theta)J_n(k_c\rho) \tag{2}$$

$$e_z(\rho,\theta)=(A\sin n\theta+B\cos n\theta)J_n(k_c\rho) \tag{3}$$

Table 1

The Values of T_{mn} for Bessel function values

ρ_{nm}	**m = 0**	**m = 1**	**m = 2**	**m = 3**	**m = 4**
**n = 1**	2.4049	3.8318	5.1357	6.3802	7.5884
**n = 2**	5.5201	7.0156	8.4173	9.7610	11.0647
**n = 3**	8.6537	10.1735	11.6199	13.0152	14.3726

The difference between the TE solution and the present solution is that the boundary conditions can now be applied directly to e_z of (3) since

$$E_z(\rho,\theta)=0, \text{ at } \rho=a \tag{4}$$

Then, know that

$$J_n(k_c a)=0, \text{ or } k_c=\rho_{nm}/a \tag{5}$$

where ρ_{nm} is the m th root of $J_n(x)$, that is $J_n(\rho_{nm})=0$. Values of ρ_{nm} are given in mathematical tables; and the propagation constant of the TM_{nm} mode is

$$\beta_{nm}=\sqrt{k^2-k_c^2}=\sqrt{k^2-(\rho_{nm}/a)^2} \tag{6}$$

And the cutoff frequency is

$$f_{c_{nm}}=\frac{k_c}{2\pi\sqrt{\mu\varepsilon}}=\frac{\rho_{nm}}{2\pi a\sqrt{\mu\varepsilon}} \tag{7}$$

Therefore, the first TM mode to propagate is the TM_{01} mode , with $\rho_{01}=2.405$ as shown in Fig. 1. TM_{01} mode is implemented on this design due to high center flux density. The understanding of transverse mode properties is significant due to the sensor behaviour.

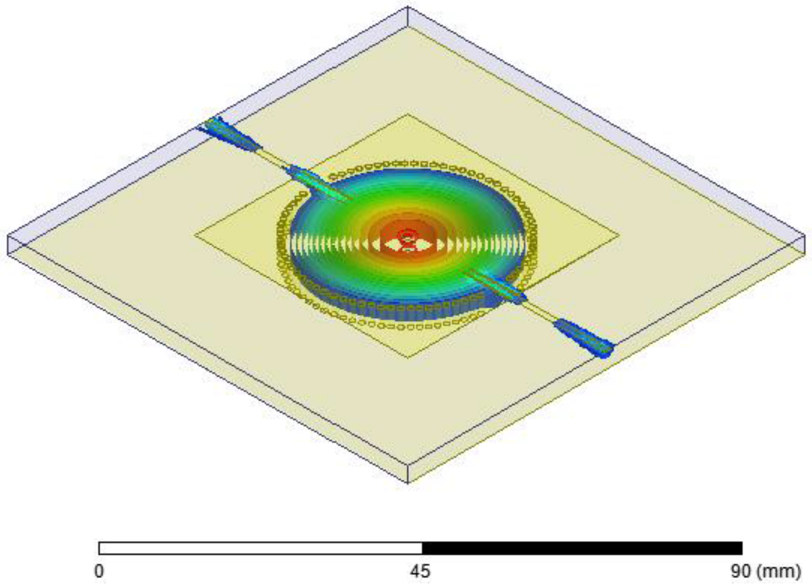

Fig. 1. The electrical flux density of the TM_{01} CSIW structure without micro capillary channel.

Supplementary Information 2

The physical dimensions of the structure are calculated based on the following relation (Pozar, 2005):

Radius of circular SIW

$$a = \frac{T_{mn} \times c}{2\pi f_r \sqrt{\varepsilon_r}} \tag{8}$$

Width of the patch

$$w = \frac{c}{2f_r}\sqrt{\frac{2}{\varepsilon_r + 1}} \tag{9}$$

Effective dielectric constant

$$\varepsilon_{eff} = \frac{\varepsilon_r + 1}{2} + \frac{\varepsilon_r - 1}{2}\left[1 + \frac{12h}{w}\right]^{-1/2} \tag{10}$$

From a practical application, via hole diameter of CSIW and its pitch, p can be determined by applying the design rules as introduced by Deslandes (Deslandes and Wu, 2003) which is shown in (11) and (12).

Diameter of via hole

$$D_v > 0.2\lambda_0 \tag{11}$$

Distance between via holes

$$\frac{D_v}{p} \leq 0.5 \tag{12}$$

Supplementary Information 3

After establishing the numerical expression for calculation of the real part permittivity and loss tangent, the imaginary part of the sample can easily be found by derivation of all those three relationships which is given as follows (Potelon et al., 2006).

$$Q_{LUT} = \frac{1}{\tan\delta}, \text{While } \tan\delta = \frac{\varepsilon''}{\varepsilon'} \quad (13)$$

Where Q_{LUT} represents the quality factor of the liquid under test, which may be calculated using the resonant centre frequency, f_c of the proposed sensor with respect to -3dB bandwidth.

$$Q_{LUT} = \frac{f_c}{BW} \quad (14)$$

The Q_{LUT} also, may be calculated using the insertion loss, (S_{21}) magnitude transmission corresponding to the resonance frequency of the loaded CSIW resonator sensor (Ansari et al., 2015).

$$Q_{LUT} = Q_U \left(1 - 10^{S_{21}/20} \right) \quad (15)$$

Where Q_U represent the quality factor under unloaded condition. Thus, the imaginary part of the proposed sensor can be calculated using (13)-(15).
